# The Sunny Side of the Network Approach to Psychopathology: Comparing Nodes as Either Problems or Strengths

**DOI:** 10.17505/jpor.2025.28094

**Published:** 2025-06-28

**Authors:** Jakob Schenström, Marie De Mey, Matthis Andreasson, Lene Lindberg, Felicia Sundström, Lars Klintwall

**Affiliations:** 1 Centre for Psychiatry Research, Department of Clinical Neuroscience, Karolinska Institutet, Sweden & Stockholm Health Care Services, Region Stockholm, Sweden, mariegdemey@gmail.com; 2 Department of Psychology, Uppsala University, Uppsala, Sweden, matthis.andreasson@psyk.uu.se; 3 Department of Global Public Health, Karolinska Institute, and Center for Epidemiology and Community Medicine Region Stockholm, Sweden, lene.lindberg@ki.se

**Keywords:** case conceptualization, positive psychology, agency, symptom networks, assessment

## Abstract

**Objectives:**

Personalized symptom networks are emerging as a tool to enhance psychiatric case conceptualizations. However, applications of the approach have so far focused on illness-causing (pathogenic) factors and their relationships with each other, whereas it is possible that a useful case conceptualization needs to include health-promoting (salutogenic) factors. The aim of this study was to investigate adolescents' and clinicians' evaluations of pathogenic and salutogenic idiographic networks.

**Methods:**

Networks were created for nine adolescent women by using the PECAN (Perceived Causal Networks) method. For every participant two networks were produced: one consisted of symptoms, such as “stuck in negative thoughts” as nodes (pathogenetic), the other health-promoting factors, such as “can let go of negative thoughts” as nodes (salutogenic). The same nine adolescents (Study I) and twenty therapists (Study II) evaluated these networks.

**Results:**

Adolescents evaluated their salutogenic networks as easier to define and create, but their pathogenic network as more useful. Therapists considered both methods to be clinically useful, but in general rated the salutogenic networks to be more informative. Both adolescents and therapists stressed the complementary use of salutogenic and pathogenic networks.

**Conclusions:**

Future studies should explore ways to integrate pathogenic and salutogenic nodes in the same network, and compare whether patients collecting longitudinal data might be differentially impacted by a focus on either symptoms or strengths.

**Practice implications:**

Person-specific networks could complement traditional case conceptualization by integrating both symptoms and resilience factors.

## Introduction

Despite extensive research, the effectiveness of psychological treatments and their impact has not improved in terms of effect sizes (Johnsen & Friborg, 2015). There is a growing focus on the high number of individuals who have multiple psychiatric diagnoses, as well as the large heterogeneity within diagnostic categories. For example, a study by Galatzer-Levy and Bryant (2013) found that there are 636,130 ways in which people can experience posttraumatic stress disorder (PTSD). While efforts have been made to develop transdiagnostic treatment protocols, some researchers are now suggesting that personalized treatments may be a more effective approach, as opposed to finding a single protocol that works for most individuals. Additionally, there is a shift away from relying solely on large Randomized Controlled Trials (RCTs) towards studying participants in a more ecologically valid manner over time, often using themselves as a control group for comparison as done in single-case experimental designs (Sahdra et al., 2024; Sundström et al., 2024; Tanious & Onghena, 2021).

The idea of tailoring treatment to an individual's specific circumstances, such as their personal history, current situation, reasons for seeking help, and current specific symptoms, is not a new concept (e.g., Paul, 1969). However, exactly how to personalize treatments is still an open question, for example recently investigated in chronic pain (Lavefjord & Sundström, 2025). One way to individualize treatments is through personal case conceptualizations, such as functional analysis (Scholten et al., 2022). These personal case conceptualizations not only inform the treating therapist, but could also provide valuable information for discussions among colleagues (Lavefjord & Sundström, 2025).

Sadly, there is little evidence supporting the idea that personalized treatments using case conceptualizations improve outcomes (Bieling & Kuyken, 2003; Ghaderi, 2006; Schulte et al., 1992; Wilson, 1996a). One possible explanation is that conceptualizations tend to have low validity, at least partly due to therapist biases (Haynes et al., 2018; Kuyken et al., 2005, Meehl, 1954). In other words, therapists may be overly influenced by previous patients or nomothetic models, i.e. seeing what they expect and therefore reducing sensitivity to the patient in front of them. Moreover, there appears to be a lack of reliability (Bieling and Kuyken, 2003) in terms of collegial consensus (Flitcroft et al. 2007).

Rather than relying on traditional case conceptualizations, a new approach called idiographic data-driven networks has been developed to enhance personalized treatment (Borsboom, 2017). This network perspective not only designs treatment plans tailored to individual patients but also seeks to address the shortcomings in the fundamental understanding of psychological diagnostics. In the traditional view, symptoms like insomnia, irritability, anhedonia and low mood are indicators of an underlying disease, in this case, depression. By contrast, the network perspective views mental disorders as a network of interconnected symptoms, with the patients' symptoms and complaints as nodes and their causal interactions as edges. According to this view, the persistence of the network is attributed to feedback loops among the edges, which can sustain psychological states regardless of triggering events.

Two methods for creating personalized symptom networks have been proposed for treatment individualization: time-series analysis of dense longitudinal data from Ecological Momentary Assessment (EMA; e.g., Levinson et al., 2021; Levinson et al., 2023; Frumkin et al., 2021; Soyster et al., 2023) and PErceived CAusal Networks (PECAN; Klintwall et al., 2023, Vogel et al, 2024). EMA networks are created using statistical estimation and require statistical assumptions (e.g., linearity and stationarity). A minimum of 50-70 data points collected over time from the participant is necessary (Bringmann et al, 2022). This allows the researcher or clinician to observe the lagged correlations between the measured symptoms. These correlations constitute the directed edges in the network, providing guidance for intervention targets (Lunansky et al., 2022). However, EMA networks have a major limitation; for example, the different symptoms interact on widely differing time spans (e.g. rumination causing sadness might act within minutes, whereas physical inactivity causes fatigue over weeks). With lag correlations, only effects on the time-scale defined by the spacing of EMA beeps can be uncovered (Bringmann et al., 2022).

PECAN is based on the idea that individuals, having experienced their symptoms, emotions and behaviors across many different situations in daily life, are able to form beliefs about cause-and-effect relationships between these phenomena (Frewen et al, 2012). Variants of PECAN include structured patient questionnaires (Klintwall et al., 2023), longitudinal daily assessments that are aggregated to yield a network (L-PECAN; Burger et al., 2024), and structured interviews (Kaariniemi et al., 2025). Therapists have found PECAN to be clinically useful (Klintwall et al., 2023), and the method has been used in clinical settings with promising evaluations by therapists and patients (Andreasson et al., 2023). Additionally, PECAN has been adapted for use with populations for whom case conceptualization might be challenging, such as adolescents (Bångstad et al., 2024).

Both time-series methods and PECAN often focus on psychopathology, i.e., creating networks on how *symptoms* interact. However, it is widely acknowledged that mental illness must always be understood in the context of healthy recovery processes and well-being (Keyes, 2009). Promoting mental health and well-being involves highlighting and strengthening protective and resilience factors. Mental illness and mental health can be seen as two separate dimensions (Keyes, 2009). Therefore, it is theorized that excluding well-being and recovery processes and factors (“salutogenic” factors) from discussions provides an incomplete picture, disregarding potential targets for intervention. Further, the repeated queries involved in EMA risks increasing patient focus on negative events and emotions (Kivelä et al, 2024), suggesting that developing methods querying patients about *what works* instead might be preferable in certain populations.

The identification of idiosyncratic salutogenic mechanisms and how they create positive feedback loops could arguably be more useful than symptoms. This could be an important complementary approach to health-promoting activities, especially for sensitive patient groups like adolescents, as it may be less aversive. Further, as most studies employing network methods (i.e. EMA and L-PECAN) to create personalized case conceptualizations rely on repeated reports by the patient, assessing positive experiences and what works might be more therapeutic and avoid an increased focus on problems in patients.

Thus, the current pilot explores these questions:

– How do adolescents experience and evaluate two versions of PECAN: one mapping their own problems (pathogenic network) and one mapping resilience factors (salutogenic network)?
– What do therapists think of the clinical utility of the resulting networks?

## Study 1

### Method

#### Recruitment and Participants

Recruitment was carried out through social media and schools in Sweden. Any adolescent who signed up for the study was eligible, the only inclusion criteria being age between 15 and 19 years. Participants were not compensated for their participation. Informed consent was collected through the website for self-referral. Participants were informed of the purpose of the study, their ability to terminate their participation at any moment during the study without having to explain why, and that they would not receive any compensation for participating beyond seeing their personalized networks. The interviewer also made a clinical evaluation of whether the participants could consent to participating. To ensure confidentiality, no identifying information was requested apart from means of contact, i.e. email address or phone number, to schedule the interviews.

Initially, 145 adolescents expressed their informed consent to participate in the study. Of these, 54 were subsequently contacted through SMS or email, resulting in responses from 17 individuals. However, out of these 17 prospective participants, four opted out beforehand, three did not attend their scheduled interview sessions, and one withdrew during the study. Thus, Study I involved nine female adolescents aged between 15 and 19. Two adolescents reported being diagnosed with autism, and one was doing a psychological evaluation for possible ADHD at the time of the interviews. Measured with RCADS, seven of the respondents self-assessed depression and anxiety over the clinical cut-off point (i.e., a *t*-value over 70), of which three had a *t*-value of 80 or higher. The median was a *t*- value of 74, with a range between 58 and 80. Several respondents mentioned psychological problems such as having/had suicidal thoughts and/or self-harm behavior during periods in life or having/had an eating disorder. Several respondents mentioned social problems such as feelings of exclusion in school and school performance stress; one respondent mentioned being bullied. One respondent mentioned severe physical illness during childhood. Seven of the respondents had some form of psychological therapy.

#### Procedure and Materials

Interested adolescents started by reading the study information and giving consent to participation. They then completed RCADS-25 (using REDcap; Harris et al, 2019), before three dates for video calls were scheduled: two meetings for the interviews and a third meeting to look at the resulting networks. Dates were scheduled so that interviews were within seven days of each other. This resulted in an average distance of 3.5 days.

*Revised Children’s Anxiety and Depression Scale (RCADS) – 25.* RCADS-25 is a self-report questionnaire comprising 25 items aimed at evaluating symptoms of depression and anxiety in children and adolescents aged 8-18. This abbreviated version (Ebesutani et al., 2012) is considered sufficiently valid and reliable for screening purposes in children and adolescents (Klaufus et al., 2020).

*Interviews: PECAN-P and PECAN-S*. The PECAN maps the respondents' perceptions of how nodes of interest are causally linked. This can then be used to create an idiographic network. This study used two PECAN variants: PECAN-P (Pathogenic) and PECAN-S (Salutogenic), with each adolescent being interviewed with both. The interviews were done in two separate video calls, with the same interviewer (author M.D.M,), in the following manner:

First, the participant selected between five and nine pathogenic items from a “menu” of common psychiatric problems. Each selected item was then specified in the words of the respondent herself (e.g., “Negative feelings” could be specified as “Anxiety”). For each selected item, respondents indicated both the unpleasantness using a 9-point Likert scale ranging from “not unpleasant” to “extremely unpleasant”, and frequency in the past month, again using a 9-point Likert scale ranging from “No days” to “All days”. Each respondent was then interviewed with PECAN, randomly either PECAN-P or PECAN-S.

In PECAN-S, the next step was rephrasing each pathogenic item into a salutogenic opposite. This was done by the interviewer asking the respondent to come up with what would be the opposite of the problem or its absence. To increase clinical utility, this rephrasing was done in such a way as to make sure the salutogenic item had a frequency in the past month higher than zero, and rephrasing was done in collaboration with the respondent to ensure this. For example, if the pathogenic item was “anxiety”, the salutogenic opposite could be “no anxiety” or “less intense anxiety” or “being able to deal with anxiety”. Each salutogenic item was then rated for frequency in the same manner as the pathogenic item.

After this “rephrasing-into-salutogenic-opposite”, the two versions of PECAN (PECAN-P and PECAN-S) were identical: in both versions, the next step was to rate every potential directed edge between the selected nodes. There are multiple ways to quantify perceived causal relations (Vogel et al., 2024). In the present study, this was done by asking respondents how frequently they experienced one factor leading to another. To be precise, the causal question was formulated as “The days you have X, does it lead to you…”, and the other items in the rephrased form specified by the respondent earlier. Each relationship was graded on a nine-point Likert scale, ranging from *Never* (0 %) to *Always* (100 %). After each interview, the experience of that interview (P or S) was evaluated. Edge strength was computed as the reported edge frequency multiplied by source-node frequency. For example, if the node “Anxiety” had a frequency of 80% and was reported to lead to “Self-harm” on 50% of occasions, the edge was given a strength of 40 %.

Rating causal relations in the two variants of PECAN took roughly the same time to complete: the PECAN-P took on average 23.5 minutes, while PECAN-S took on average 24.7 minutes.

*Visualization of networks.* Two networks were created for each respondent: one pathogenic (red nodes) and one salutogenic (green nodes). These used a Fruchterman-Reingold force-directed layout and showed only the strongest edges (the number of edges corresponding to the number of nodes in the network). In the third and final video call, the respondent saw her networks and evaluated them. *Evaluation questionnaires*. After completing PECAN-P and PECAN-S each, the respondent ranked five statements evaluating the interview, using a five-point Likert scale, ranging from 1 (Don't agree at all) to 5 (Agree completely). The statements were: “*The interview was too long*”, “*The questions made sense, and my answers were well thought out”*, “*The interview made me pessimistic about my future*”, “*The interview made me optimistic about my future*” and “*The interview helped me understand my life better*”.

When presented with the two networks in the final third video call, the respondents evaluated the networks using a 5-point Likert scale: “Not”, “A bit”, “Quite”, “Very”, and “Completely” on the following five questions: 1) “How clear is the network?”; 2) “How informative is this network?” 3) “How motivating is this network?” 4) “How logical is this network?”, 5) “How easy to understand is this network?”. Finally, the respondent was asked: "Which network do you prefer?”

### Results

#### How do adolescents experience and evaluate two versions of PECAN: one mapping their own problems (pathogenic network) and one mapping resilience factors (salutogenic network)?

**Figure 1:**
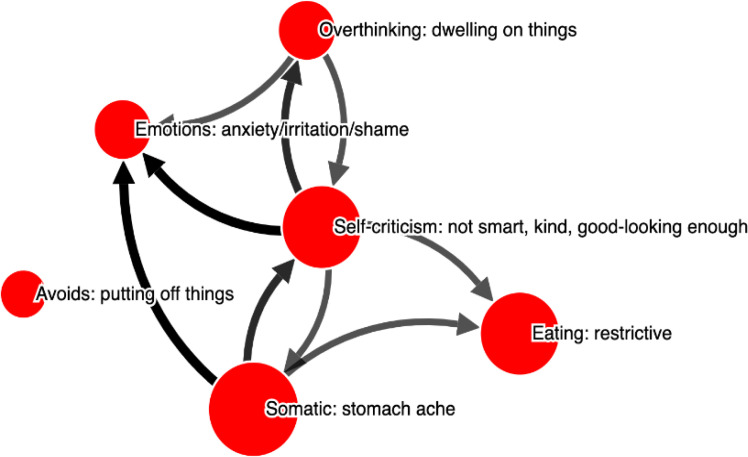
Example of an adolescent’s pathogenic network. Node labels show **Category: Specification**, i.e., the problem area and then the specification the adolescent provided in her own words

**Figure 2:**
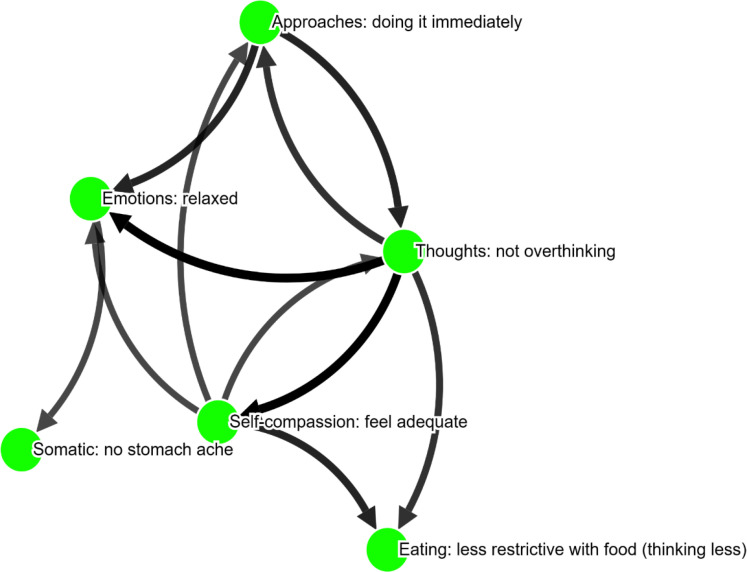
Example of an adolescent’s salutogenic network. Node labels show **Category: Specification**, i.e., the problem area and then the specification the adolescent provided in her own words

**Figure 3:**
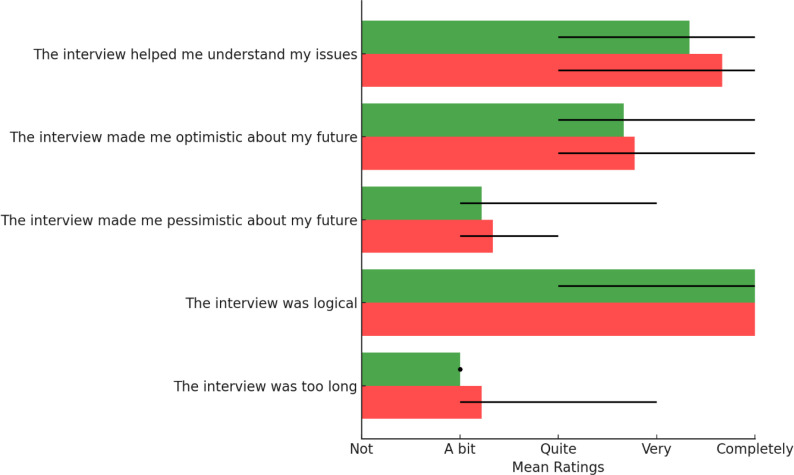
Adolescents’ evaluation of the interviews, for salutogenic interviews (PECAN-S; green) and pathogenic (PECAN-P; red). Whiskers denote min-max

**Figure 4:**
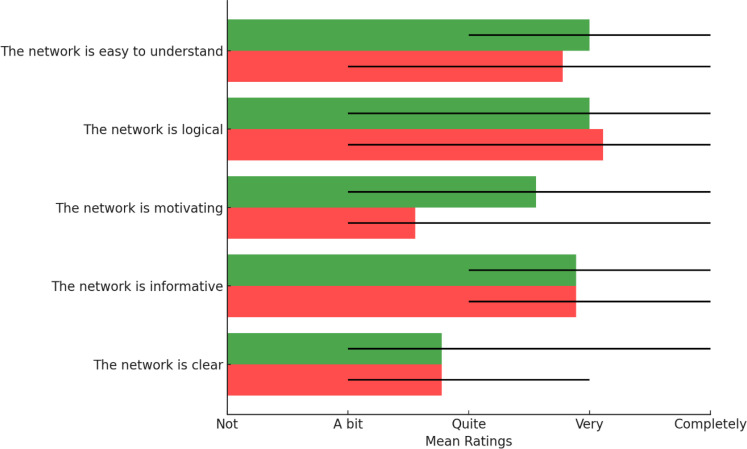
Adolescents’ evaluation of their networks (salutogenic network in green, pathogenic network in red). Whiskers denote min-max

Figures 1 and 2 depict the two person-specific networks for one adolescent, to illustrate how these networks looked like when presented to adolescents themselves. In Figures 3 and 4 we see the average evaluations made by adolescents, rating the two versions of the interviews and resulting networks. As can be seen, the interviews and networks were rated very similarly

## Study 2

### Method

#### Recruitment and Participants

To evaluate the clinical utility of salutogenic and pathogenic networks, therapists were recruited using convenience sampling in Sweden through social media, email lists, and collegial recruitment. Therapists received gift cards worth 10 euros. Twenty therapists completed the survey.

The clinical experience of working with adolescents in the sample ranged from 1 to 13 years (*M* = 3.7, *SD* = 3.3), a majority worked with CBT-based treatments (90%), while only 30 % indicated that they worked with treatments using a psychodynamic approach. The alternatives were not mutually exclusive. Regarding familiarity with networks, therapists scored a mean of 6.1 (*SD* 2.0) on a scale of 1 to 10 indicating to what extent they perceived networks as familiar in the context of clinical psychology.

#### Procedure and Materials

After reporting background information, therapists were introduced to network theory and PECAN through a five-minute introductory video. After the video, they were presented with all the nine participants’ networks from Study I, one by one. The networks were presented in pairs consisting of the salutogenic and pathogenic network for each adolescent, resulting in a total of 18 networks. The order of the participants was randomized, and whether the salutogenic or the pathogenic network was shown first was also randomized. To ensure they properly processed the networks, for each network, the therapist was asked to pick a treatment target from the network and motivate this choice.

After the therapists had processed all networks, a concluding set of questions were asked for the whole body of networks (all answered on a 5-point Likert scale): “How logical were the networks?”, “How much information did the networks give you about the adolescents’ condition?” and “How comprehensible were the networks?”. The therapists were also asked to evaluate the two types of networks in a freetext question.

## Results

### What do therapists think of the clinical utility of the resulting networks

#### Pathogenic network

Exemplified with the same adolescent shown above (Figure 1), the responses from the therapists were as follows:

– The need to address self-criticism using compassion-focused therapy and other cognitive interventions. The directed edge from self-criticism to anxiety, procrastination, and stomach pain suggests it’s a central node affecting various aspects of the patient’s well-being. This was mentioned by 60% of therapists.
– Therapists mentioned a need to explore somatic symptoms, specifically stomach pain, through medical assessments alongside psychological interventions. There was a concern to differentiate whether the stomach issues are primarily somatic or psychosomatic, suggesting interventions like interoceptive exposure or regular eating patterns. Mentioned by 53 %.
– Concern with how cognitive patterns like rumination impact the patient's functioning. Suggestions included behavior analysis and cognitive restructuring to manage these thought processes, linking them to broader symptoms like anxiety and procrastination. Mentioned by 33 %.

When asked about which node to target in treatment, “Self-criticism” was mentioned most frequently (63 %), followed by “Somatic concerns” (26 %), “Emotions” (26 %) and “Overthinking” (26 %).

#### Salutogenic network

Exemplified with the same adolescent shown above (Figure 2), the responses from the therapists were as follows:

– The therapist found the network useful as it promotes engagement and immediate action. This was seen as aligning with behavioral activation strategies. However, a limitation is that this may not address deeper cognitive or emotional issues unless combined with other therapeutic approaches. This was mentioned by 36 % of therapists.
– Focusing on thought processes and self-compassion is crucial for addressing core cognitive distortions and emotional vulnerabilities, making it a valuable component of strength-based therapies. This approach fosters resilience and positive self-regard, building a sustainable foundation for mental health. Implementing it can be challenging if patients have deeply ingrained negative self-concepts and require ongoing effort. This was mentioned by 36 % of therapists.
– How well the strength-based model applies to the patient's situation, ensuring the interventions are effective and realistic. Such critical evaluation is necessary to confirm that the model doesn't merely idealize strengths but actively leverages them in therapy. This was mentioned by 21% of therapists.

When asked which node to target in therapy, the most commonly selected node was “Approaches” (37 %) followed by “Not overthinking” (32 %) and “Self-compassion” (21 %). However, 26 % of therapists were not sure how to pick a target node in the salutogenic network for this adolescent.

**Figure 5:**
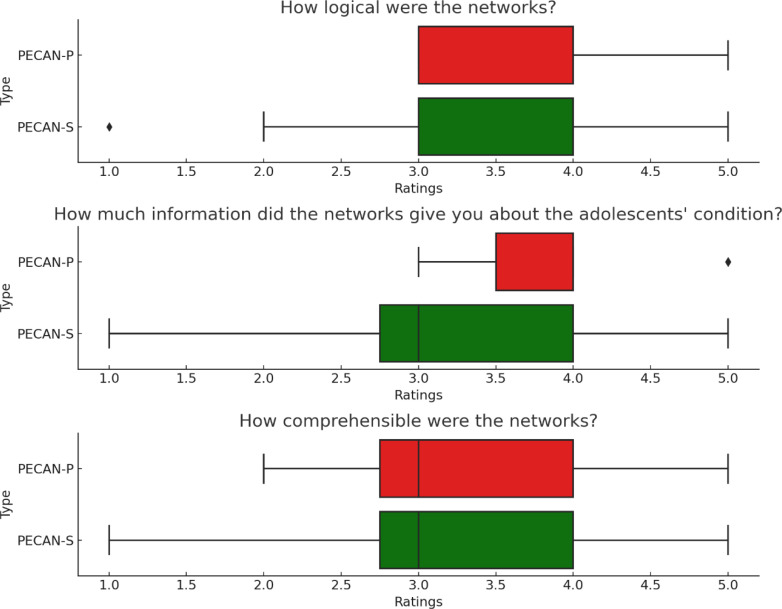
Therapist evaluations of the two types of networks as a whole

After having reviewed all networks from all participants, both pathogenic and salutogenic, therapists evaluated the two types quantitatively (Figure 5) and provided general feedback to the two types. Three recurring themes were found:

*Both networks are complementary and necessary (40%).* Many respondents highlighted that salutogenic and pathogenic networks serve complementary purposes. Salutogenic networks provide hope and motivation, while pathogenic networks focus on identifying and addressing difficulties. Both are necessary for a holistic understanding and effective intervention planning. Example quote: “Both are needed to understand and move forward. Strengths provide positivity and hope, while a network of problems identifies difficulties and guides interventions.”

*Strength networks provide unique insights (25%).* Salutogenic networks were seen as valuable for understanding what promotes well-being and identifying areas to reinforce. They add a positive perspective to therapy and will increase patient motivation. Example quote: “A network of strengths contributes positivity and hopefulness, which I believe is helpful for increasing motivation in treatment. It’s not just about alleviating issues but reinforcing what works well.”

*Problem networks are more actionable (20%).* Problem networks were typically seen as more concrete and easier to use for intervention planning. They were viewed as better suited for addressing immediate challenges. Example quote: “It was easier to think about interventions based on the symptoms. They clearly show what needs to be addressed to create change.”

## Discussion

The present paper aimed to investigate adolescents’ and clinicians’ evaluations of pathogenic and salutogenic idiographic networks. Study 1 focused on creating and evaluating pathogenic and salutogenic networks created by adolescents. Study 2 addressed therapists’ perceptions of the adolescent networks that were the results of Study 1.

Results from Study 1 show that adolescents perceived both types of interviews and networks as logical and helpful in understanding their problems. In evaluating their networks, most participants rated them as quite easy to understand and logical, irrespective of network type. A deviation of this positive pattern was found in how clear the network was found to be, with most participants perceiving them as ‘a bit’ or ‘quite’ clear, with the pathogenic networks being perceived as less clear.

Results from Study 2 mirror the findings from Study 1. Both types of networks were perceived as logical, informative, and comprehensible by the therapists. Therapists stressed the complementary roles of salutogenic and pathogenic networks, preferring to have both for a client.

These findings align with previous research on patients’ and therapists’ perceptions of networks, showing that both groups perceive perceived causal networks as helping them understand and identify interconnected factors contributing to a patient’s state (Andreasson et al., 2023; Klintwall et al., 2023). What is new about the current study is that it focused on adolescents, included both pathogenic and salutogenic networks and compared the utility of these, something not previously done.

Our results contrast with earlier findings showing that therapists are often skeptical of networks based on EMA data (see for example Hall et al., 2022). This discrepancy may be explained by differences in methodology. PECAN networks are based on patients’ perceived causal beliefs rather than statistical associations, and where EMA networks are often underpowered and thus very sparse, PECAN networks are typically quite dense. Although not a formal comparison, the present study is an indication that both patients and therapists might find networks created from PECAN as more useful.

Since both pathogenic and salutogenic networks were perceived as fairly easy to understand and helpful for participants, the choice between the two could be made on a case-by-case basis. Since this is the first study to compare the utility of salutogenic and pathogenic networks, both generalizations and clinical applications based on the results from the current study should be made with caution.

The study has several limitations that should be mentioned. Firstly, the sample in both studies can be considered too small to generalize findings to any population or to enable a statistical comparison between the ratings of the two networks. It is possible that a larger sample would find differences between the PECAN-S and PECAN-P interviews and resulting networks. Secondly, the sample in Study 1 was exclusively female and limited to adolescents. One or the other of the two types of networks is likely preferred for particular patient populations (e.g., for fatigue patients it might not be advisable to reinforce a focus on fatigue symptoms in the patient, and thus a salutogenic focus is to prefer), which should be investigated further. Thirdly, the sample in Study 2 can be seen as an artificial sample because the therapists did not know the adolescents. In realistic clinical settings, networks would be used as part of a clinical case conceptualization for a client the therapist might know quite well.

An important implication of our findings is the often underestimated role of the patient as a knowledgeable partner in understanding their own difficulties. Traditional case conceptualizations and data-driven network models tend to prioritize clinician input or statistical associations, which may overlook the rich experiential knowledge patients bring. PE-CAN offers a structured way to tap into these “personal data”: the accumulated experiences patients have about what tends to follow what in their lives. By explicitly mapping perceived causal relationships between behaviors, symptoms, and life events, PECAN makes use of this internal dataset. This participatory model may not only increase the validity of case conceptualizations but also enhance patient engagement, ownership, and therapeutic alliance. Future studies could explore the possibility to combine PECAN (whether salutogenic or pathogenic) with more objective methods to create person-specific networks (for example, see Scholten et al, 2025; Bellander et al, 2025) to increase validity and clinical utility.

From a psychotherapeutic point of view, the nodes in the salutogenic networks created in the study are unnecessarily limited by being directly linked to the pathogenic nodes (salutogenic nodes were created as opposites of the pathogenic network). For example, if an adolescent had problems with worrying thoughts, the corresponding salutogenic node could be rephrased as “letting go of thoughts”. Perhaps a more useful approach would be to create the salutogenic network independently of the pathogenic, i.e., selecting completely new nodes. Even if a patient does not have loneliness problems, having a salutogenic node such as “Hanging out with friends” could still make sense. This is something that should be explored in future studies.

Perhaps the difference between a salutogenic or pathogenic focus is more relevant to other, longitudinal, network methods (EMA and L-PECAN). In these methods, patients are asked to rate nodes several times per day, sometimes more than hundreds of assessments in order to create networks. One can easily imagine that this leads to an increased focus on problems and negative emotions, which is not only aversive to the patient but might in fact increase symptom load. Future studies could compare whether collecting longitudinal salutogenic data in contrast helps the patient savour positive experiences and decrease a focus on symptoms.

### Conclusions

Our study demonstrates that both salutogenic and pathogenic networks can be perceived as clinically beneficial, serving different functions. Future studies should explore whether both sides can be incorporated in one network, while at the same time avoiding creating a network that is too complex and thus clinically less applicable.

### Practical implications

The methods developed within the network approach to psychopathology promise to improve quality in individual case conceptualizations. Whereas network studies typically focus on patient symptoms and complaints, the present study explored the possibility of complementing this with networks of patient strengths. Results showed that patients and therapists viewed these two types of networks as complementary. Future research should investigate if this holds in a larger sample within a clinical setting and what the benefits in treatment might be.

## Data Availability

Data and redcap questionnaires are available upon request from the corresponding author.

## Action editor

Lars-Gunnar Lundh served as action editor for this article.

## Open access

This article is distributed under the terms of the Creative Commons Attribution 4.0 International License (http://creativecommons.org/licenses/by/4.0/), which permits unrestricted use, distribution, and reproduction in any medium, provided you give appropriate credit to the original author(s) and the source, provide a link to the Creative Commons license, and indicate if changes were made.
